# Meeting report from the 2nd International Symposium on New Frontiers in Cardiovascular Research. Protecting the cardiovascular system from ischemia: between bench and bedside

**DOI:** 10.1007/s00395-015-0527-0

**Published:** 2015-12-14

**Authors:** Hector A. Cabrera-Fuentes, Corina Alba-Alba, Julian Aragones, Jürgen Bernhagen, William A. Boisvert, Hans E. Bøtker, Gabriela Cesarman-Maus, Ingrid Fleming, David Garcia-Dorado, Sandrine Lecour, Elisa Liehn, Michael S. Marber, Nephtali Marina, Manuel Mayr, Oscar Perez-Mendez, Tetsuji Miura, Marisol Ruiz-Meana, Eduardo M. Salinas-Estefanon, Sang-Bing Ong, Hans J. Schnittler, Jose T. Sanchez-Vega, Adriana Sumoza-Toledo, Carl-Wilhelm Vogel, Dina Yarullina, Derek M. Yellon, Klaus T. Preissner, Derek J. Hausenloy

**Affiliations:** Institute of Biochemistry, Medical School, Justus-Liebig University, Giessen, Germany; Cardiovascular and Metabolic Disorders Program, Duke-National University of Singapore, Singapore, Singapore; National Heart Research Institute Singapore, National Heart Centre Singapore, Singapore, Singapore; Department of Microbiology, Kazan Federal University, Kazan, Russian Federation; Escuela de Ingeniería y Ciencias, Centro de Biotecnología-FEMSA, Tecnológico de Monterrey, Monterrey, NL México; Institute of Genetics, Univeristy of the Sea. Puerto Escondido Campus, Oaxaca Oaxacan System of State Universities (SUNEO), Oaxaca, México; Research Unit, Hospital of Santa Cristina, Research Institute Princesa (IP), Autonomous University of Madrid, Madrid, Spain; Institute of Biochemistry and Molecular Cell Biology, RWTH Aachen University, Aachen, Germany; Center for Cardiovascular Research, John A. Burns School of Medicine, University of Hawaii, Honolulu, USA; Department of Cardiology, Aarhus University Hospital, Skejby, Aarhus N, Denmark; Department of Hematology, Instituto Nacional de Cancerología, Mexico City, Mexico; Institute for Vascular Signalling, Centre for Molecular Medicine, Goethe-University, Frankfurt, Germany; Valld’Hebron University Hospital and Research Institute, Barcelona, Spain; Hatter Institute and MRC Inter-University Cape Heart Unit, Faculty of Health Sciences, University of Cape Town, Cape Town, South Africa; Institute for Molecular Cardiovascular Research, RWTH University Hospital Aachen, Aachen, Germany; Department of Cardiology, The Rayne Institute, St Thomas’ Campus, King’s College London, London, UK; Department of Clinical Pharmacology, University College London, London, UK; The James Black Centre, King’s College, University of London, London, UK; Department of Molecular Biology, National Institute of Cardiology, Mexico City, Mexico; Department of Cardiovascular, Renal and Metabolic Medicine, Sapporo Medical University School of Medicine, Sapporo, Japan; Laboratorio de Biofísica Cardiaca, Instituto de Fisiología, Universidad Autónoma de Puebla, Puebla, Mexico; Institute of Anatomy and Vascular Biology, Westfalian-Wilhelms-University, Münster, Germany; Laboratory of Parasitology, Department of Microbiology and Parasitology, Faculty of Medicine, Universidad Nacional Autónoma de México, Mexico City, Mexico; Laboratorio Multidisciplinario de Ciencias Biomédicas, Instituto de Investigaciones Medico-Biológicas, Universidad Veracruzana campus Veracruz, Veracruz, Mexico; Department of Pathology, John A. Burns School of Medicine, University of Hawaii, Honolulu, USA; The Hatter Cardiovascular Institute, University College London, London, UK; The National Institute of Health Research University College London Hospitals Biomedical Research Centre, London, UK

**Keywords:** Atherosclerosis, Cardioprotection, Cardiovascular disease, Endothelial permeability, Extracellular RNA, High-density lipoprotein, Hypoxia, Inflammation, Ischemia/reperfusion injury, Macrophage polarization, MicroRNAs, Mitochondria, Platelet dysfunction, Vascular biology

## Abstract

Recent advances in basic cardiovascular research as well as their translation into the clinical situation were the focus at the last “New Frontiers in Cardiovascular Research meeting”. Major topics included the characterization of new targets and procedures in cardioprotection, deciphering new players and inflammatory mechanisms in ischemic heart disease as well as uncovering microRNAs and other biomarkers as versatile and possibly causal factors in cardiovascular pathogenesis. Although a number of pathological situations such as ischemia–reperfusion injury or atherosclerosis can be simulated and manipulated in diverse animal models, also to challenge new drugs for intervention, patient studies are the ultimate litmus test to obtain unequivocal information about the validity of biomedical concepts and their application in the clinics. Thus, the open and bidirectional exchange between bench and bedside is crucial to advance the field of ischemic heart disease with a particular emphasis of understanding long-lasting approaches in cardioprotection.

## Introduction

Ischemic heart disease (IHD) is the leading cause of death and disability worldwide [104]. Thus, new treatment strategies are required to protect the heart from the detrimental effects of acute ischemia/reperfusion injury (IRI) so as to reduce myocardial injury, to preserve left ventricular systolic function, and to prevent the onset of heart failure [40]. During the 2nd International Symposium on “New Frontiers in Cardiovascular Research” (Huatulco-Oaxaca, Mexico), basic researchers and clinicians discussed new biomedical developments as well as novel targets and respective interventional strategies in the areas of heart failure, inflammatory mechanisms, and cardioprotection. In essence, the meeting covered heterogenous and unrelated intra- as well as extracellular molecular targets such as cytokines, ion channels, extracellular nucleic acids, or mitochondrial factors, which are all linked to the development or prevention of IHD, which not only reflect the complexity of the biological system but also indicate the variety of possible interventional approaches that can be helpful or even lifesaving as a cardioprotective strategy.

## The challenges of translating cardioprotection into the clinical setting

Derek Yellon (UK) opened the meeting by providing an expert overview of cardioprotection from bench-to-bedside with focus given to new therapeutic targets for cardioprotection and the challenges facing the translation of new cardioprotective therapies from the laboratory to the clinical arena; a topic which has been extensively discussed in recent literature, and which has been attributed to a number of factors including the use of inappropriate animal models and poor clinical trial design (reviewed in [27, 29, 33, 52]). In particular, the influence of co-morbidities (e.g., diabetes, hypertension, and hyperlipidemia) and concomitant medication (nitrates, volatile anesthetics, and propofol) on cardioprotection is important factors to take into consideration, especially given the wealth of preclinical data suggesting that these factors confound cardioprotection [20].

The heart can be protected from acute IRI, both experimentally and in the clinical setting, by subjecting it to brief episodes of ischemia and reperfusion, a phenomenon termed “ischemic preconditioning” [72]. However, this manipulation requires an invasive strategy applied directly to the heart and also necessitates that the intervention is applied prior to the index ischemic episode, which of course is not possible in patients presenting with an acute myocardial infarction (MI). Here, the intervention by remote ischemic conditioning (RIC), a noninvasive, low-cost, easily administered cardioprotective procedure, can be applied after the onset of myocardial ischemia and, therefore, has therapeutic potential for patients with ST-segment elevation myocardial infarction (STEMI), treating by primary percutaneous coronary intervention (PPCI) [31, 37, 78, 93] or thrombolysis [107]. RIC can be delivered by inflating a standard blood pressure cuff placed on the upper arm or thigh, to induce brief cycles of ischemia and reperfusion to the arm or leg. Whether RIC has the potential to improve short-term clinical outcomes [71, 94, 101] and to prevent long-term major adverse cardiac events in this patient group is currently being investigated by Hans Botker and Derek Hausenloy in the CONDI2/ERIC-PPCI trial, a 4300 STEMI patient international (Denmark, UK, and Spain), multicentre randomized controlled clinical trial by investigating whether RIC can reduce the rates of cardiac death and hospitalization for heart failure at 12 months (ClinicalTrials.gov Identifiers: NCT01857414 and NCT02342522). Interestingly, Botker’s group has found that certain co-morbidities (age, diabetes, hypertension, high body mass index, hyperlipidemia, and left ventricular hypertrophy), and concomitant medications (β-blocker, ACE-inhibitor, calcium antagonists, and statins) did not appear to affect the cardioprotective efficacy of RIC in reperfused STEMI patients. Although there is a wealth of preclinical data indicating that co-morbidities and concomitant medication can confound endogenous cardioprotection much of the evidence is restricted to ischemic preconditioning and postconditioning as opposed to remote ischemic conditioning [20].

The results of the recently published ERICCA [28] and RIPHeart [67] multicentre clinical trials have raised the issue of whether concomitant medication can interfere with the cardioprotective effects of RIC in patients undergoing cardiac surgery. In these large clinical outcome studies, RIC using transient arm ischemia/reperfusion was found to have no effect in terms of reducing perioperative myocardial injury or improving short-term or long-term major adverse cardiovascular events. It has been suggested that the use of propofol anesthesia may have, in part, attenuated the cardioprotective effects of RIC in the setting of cardiac surgery [48, 49], although the mechanism for this interaction remains unclear and needs to be investigated [38]. The role of propofol in cardioprotection is quite complex and, at times, the data appear inconsistent. A number of experimental studies [54, 59] and even one clinical study in cardiac surgery [87] have found propofol to protect the myocardium against acute ischemia/reperfusion injury through an antioxidant effect and inhibition of MPTP opening [42]. Moreover, there is no clear relationship between propofol use and the cardioprotective efficacy of RIPC, with several clinical studies demonstrating RIPC cardioprotection despite the presence of propofol, and others finding no benefit with RIPC, even in the absence of propofol.

## Mitochondria as targets for cardioprotection

Mitochondrial dysfunction lies at the heart of a number of cardiovascular diseases including acute IRI, and treatments aimed at preserving mitochondrial function represent an important cardioprotective strategy for limiting MI size and preserving cardiac function [36]. Glycogen synthase kinase-3β (GSK-3β), a constitutively active multifunctional kinase, is an important downstream target of a number of pro-survival signaling pathways, recruited by cardioprotective strategies, such as ischemic conditioning, as it contributes to IRI by increasing the susceptibility to mitochondrial permeability transition pore (MPTP) opening [100]. The interplay between GSK-3β and mitochondria in the context of acute myocardial IRI was addressed by Tetsuji Miura (Japan) by showing that the mitochondrial translocation of GSK-3β by oxidant stress appears to be mediated by its kinase-dependent interaction with the voltage-dependent anion channel 2 and that translocated GSK-3β interacted with complex III of the electron transport chain, leading to increased cytotoxic reactive oxygen species (ROS) production [99, 100]. Although the therapeutic inhibition of the mitochondrial translocation of GSK-3β may provide a promising approach to cardioprotection without disturbing the physiological functions of GSK-3β, certain co-morbidities such as diabetes, hypertension, and chronic renal failure can disrupt cytoprotective signaling pathways of GSK-3β at different sites, and this may contribute, in part, to the heightened harmful effects of acute myocardial IRI under these medical conditions.

The phenomenon of “mitochondrial dynamics” was introduced by Derek Hausenloy (UK and Singapore) as a novel target for cardioprotection—this refers to the ability of mitochondria to move and change shape by undergoing fission and fusion to generate fragmented and elongated mitochondria, respectively [26, 74, 75]. These processes are required to maintain healthy mitochondria and normal cell function, and their genetic or pharmacological inhibition offer a potential cardioprotective strategy [76]. In particular, the manipulation of mitochondrial fusion proteins such as mitofusin 2 (Mfn2) in the adult heart produced unexpected results in terms of cardioprotection [17]. This fusion protein has been shown to act as a tether between the sarcoplasmic reticulum (SR) and mitochondria [16] and thereby facilitates calcium signaling between these two organelles as a critical step in the coupling of mitochondrial energy production with the energy requirements of the contractile system. Cardiac-specific deletion of the mitochondrial fusion proteins Mfn1 and Mfn2 in the adult murine heart resulted in reduced infarct size when compared to wild-type littermates. This cardioprotective effect appeared to be mediated by the dissociation of the SR from mitochondria, thereby disrupting calcium signaling between both organelles and decreasing mitochondrial calcium overload, attenuating oxidative stress and decreasing MPTP opening. Recent data indicate that the mitochondrial fusion and fission proteins appear to provide therapeutic targets in heart failure, pulmonary hypertension, or cardiomyocyte stem cell differentiation as well [26, 75, 78]. Yet, based upon their pleiotropic functions, manipulation of these mitochondrial fusion and fission proteins needs to be precisely defined in each acute setting in order to prevent detrimental off-target effects such as cardiomyopathy.

The impact of advanced age on mitochondrial dysfunction, which occurs in the context of acute myocardial IRI, was discussed by Marisol Ruiz-Meana (Spain). Preclinical data have suggested that mitochondria play a causative role in the increased susceptibility of the senescent myocardium to ischemic damage. The existence of an age-dependent disruption of the molecular communication between SR and mitochondria appears to have consequences on calcium handling and bioenergetics in response to acute myocardial IRI [22]. Moreover, oxidation of mitochondrial ATP synthase aggravated mitochondrial membrane permeabilization and cell death in the first minutes of reperfusion [21]; yet, the underlying mechanisms remain to be characterized with regard to the response to injury and cardioprotection in the aging heart, representing a highly relevant topic considering our aging population.

Finally, translating a therapeutic cardioprotective approach that targets mitochondria into the patients´ benefit has recently been illustrated with the neutral results of the CIRCUS trial: This trial failed to demonstrate improved clinical outcomes at 1 year (endpoints: cardiac death, heart failure, and adverse left ventricular remodeling) in STEMI patients who received an intravenous bolus of the MPTP inhibitor cyclosporine-A prior to PPCI [14]. Although the reasons for the failure of this trial are not clear, a number of factors should be taken into consideration: (1) Not all experimental studies investigating the infarct-limiting effects of CsA have been positive [44, 60], and the clinical data supporting its cardioprotective effects in STEMI patients have been limited; (2) In the CIRCUS trial, a novel formulation of cyclosporine called CicloMulsion was used, whereas Sandimmune was used in the original study [79]—the intralipid carrier vehicle used in CicloMulsion has been reported in experimental studies to be cardioprotective [53], and therefore its presence may have diminished a difference between CsA and vehicle control; (3) Although the initial positive proof-of-concept study only recruited patients treated by direct stenting to ensuring abrupt reperfusion without any angioplasty pre-dilatation [79], the CIRCUS trial also recruited STEMI patients who had not been directly stented [14, 32, 34].

## Combination therapy as a cardioprotective strategy

While several single-target treatment strategies failed to improve clinical outcomes in patients with ischemic heart disease [29, 33, 52], the concept of combination therapy as a potentially more effective approach for preventing myocardial reperfusion injury was introduced by David Garcia-Dorado (Spain). He has investigated the MI-limiting effects of combining ischemic postconditioning with a pharmacological cardioprotective strategy using an in vivo porcine model of acute IRI [83]. Based on the cardioprotective effects of glucagon-like peptide-1 (GLP-1) [30] or its analogs (such as exenatide) [61, 103], administered at the onset of reperfusion, their combination with RIC resulted in a significant reduction of infarct size as compared to the individual treatments alone and appeared to be mediated through two distinct pro-survival pathways [4]. In the COMBinAtion Therapy in Myocardial Infarction (COMBAT-MI) trial (ClinicalTrials.gov Identifier: NCT02404376), STEMI patients treated by PPCI will be subjected to this combined therapy, aiming to protect against myocardial infarction and prevent the onset of heart failure.

## High-density lipoprotein and cardiovascular disease

Low levels of high-density lipoprotein (HDL) cholesterol constitute a major risk factor for cardiovascular disease; however, recent therapies aimed at raising HDL levels have failed—the underlying reasons remain unclear. Native and/or synthetic HDL-subtypes were studied in experimental models of IHD to characterize their possible role in cardioprotection, and Sandrine Lecour (South Africa) reported that HDL subtype 3, containing a high level of sphingosine-1 phosphate, may be superior against acute IRI than HDL subtype 2 [7], indicating that it is the quality of HDL rather than its overall quantity that confers the cardiovascular benefits [84–86].

The close proximity of epicardial adipose tissue toward the media of arteries and its colocalization with atheroma lesions commonly observed in patients submitted to revascularization strongly suggests an active role of epicardial adipose tissue in the genesis and progression of atherosclerosis [63]. Epicardial adipose tissue may thereby act as a paracrine organ in the induction of inflammation and the regulation of the metabolism of vascular smooth muscle cells. Based on clinical studies, the contribution of epicardial adipose tissue and HDL in atheroma calcification of coronary arteries was proposed by Oscar Perez-Mendez (Mexico) [24]. Here, an altered mRNA-expression of osteopontin, osteonectin, and osteoprotegerin was observed in epicardial adipose tissue obtained from patients with coronary artery disease submitted to revascularization surgery as compared to control individuals who underwent aortic replacement without MI. Since the expression changes of these three genes were clearly associated with the indicated HDL subclasses, a potential role of HDL in the regulation of atheroma calcification appears likely.

## Inflammation in myocardial infarction and subsequent left ventricular remodeling

Atherosclerosis is a chronic inflammatory disease and is one of the major underlying causes of an acute myocardial infarction [55]. At the meeting, Jürgen Bernhagen (Germany) summarized the wealth of evidence supporting the pro-atherogenic effects of the chemokine-like cytokine macrophage migration-inhibitory factor (MIF) and highlighted emerging novel data reporting its cardioprotective activities in the setting of acute myocardial IRI. His presentation reviewed the distinct mechanistic differences between MIF’s pro-atherogenic action in the vasculature (CXCR2/4-mediated leukocyte recruitment, vascular inflammation, and plaque destabilization) versus its cardioprotective action in the ischemic heart (CD74/AMPK- and S-nitrosylated MIF-mediated cardioprotection). From a therapeutic perspective, blocking MIF to prevent atherogenesis may adversely block the cardioprotective effect of MIF and should be used with caution. His research group has identified CXCR7 as a novel, fourth, MIF receptor [3, 12]. Finally, further translational considerations were discussed on the role of the broader MIF protein family in clinical outcomes of cardiac surgery patients [96]. Furthermore, upon myocardial infarction, cytokines, such as tumor-necrosis-factor α (TNF-α) as well as alarmins, particularly extracellular RNA (eRNA) become released or liberated from the injured cardiac tissue and represent mediators of acute myocardial IRI [10, 11], as reported by Klaus T. Preissner (Germany). In fact, patients subjected to acute global IRI during cardiac bypass surgery and exhibited significant elevation of plasma eRNA and TNF-α [9]. In experimental models, including in vivo murine myocardial IRI or the isolated Langendorff-perfused rat heart, as well as in cardiomyocytes subjected to hypoxia, eRNA promoted TNF-α liberation through the activation of TNF-α converting enzyme (TACE) [10]. Conversely, TNF-α promoted further eRNA release especially under hypoxia, thereby feeding a vicious cell damaging cycle during IRI with the massive production of oxygen radicals, mitochondrial damage, decrease in antioxidant enzymes, and decline of cardiomyocyte functions (unpublished data). The administration of RNase1 or the TACE-inhibitor TAPI prevented cell death and significantly decreased myocardial infarction. This regimen allowed the reduction in cytokine release, normalization of antioxidant enzymes as well as preservation of cardiac tissue. Finally, a dramatic increase of endogenous vascular RNase1 in human subjects was achieved by inducing remote ischemic preconditioning [37], a noninvasive intermittent limb ischemia and reperfusion using a simple external occluder, thereby proving the impact of the eRNA/RNase system in remote ischemic preconditioning [9].

The left ventricular (LV) remodeling, which takes place following an acute MI, is a critical determinant of final LV systolic function, the onset of heart failure and adverse clinical outcome. In this regard, Elisa Liehn (Germany) discussed the role of immune cells which are able to modify collagen synthesis [56–58] and to activate different signaling pathways in myofibroblasts, with consequences on tissue healing, scar formation, and heart function after an acute MI [15, 43, 89]. In particular, neutrophils activate inflammatory- (TGF-ß1, IL-1ß), differentiation-, migration-, proliferation- and angiogenesis-related genes, thus maintaining a soft cytoskeletal structure, whereas monocytes activate inflammatory- (angiotensin II, NFκB), apoptosis- and remodeling-related genes, thereby increasing the stiffness of the fibroblasts. As a consequence, targeting the extracellular matrix to prevent LV remodeling post-MI appears to be a promising therapeutic approach to provide synergistic benefits with cardiomyocyte-directed therapies.

## Cardiac myosin binding protein C: a novel biomarker for myocardial injury

The ability to detect the presence of myocardial necrosis in patients presenting with chest pain as a criterion for diagnosing an acute MI depends on the ability to accurately quantify the extent of damage to the myocardium [102]. The use of increasingly sensitive assays for the cardiac forms of Troponin I (cTnI) and Troponin T (cTnT) has revolutionized the care of patients presenting with suspected non-ST elevation acute coronary syndromes (NSTE-ACS) [70]. However, troponins are released slowly after cardiac injury, and it is therefore necessary to take heed of their exceedingly low concentrations. Consequently, sensitivity is achieved but at the expense of poor specificity [47]. Following a systematic screen of proteins that appeared in the coronary sinus after mild myocardial infarction, Michael Marber (UK) presented the cardiac restricted Cardiac Myosin Binding Protein C (cMyC) as a potential new biomarker [41] with faster release and clearance kinetics than the troponins [6]. The different dynamics of this novel biomarker would be expected to translate into improved diagnostic performance for the detection of acute MI in NSTE-ACS, a hypothesis which is currently being tested in a large unselected cohort of such patients [25].

## Cellular adaptation to hypoxia in cardiovascular disease

Different endogenous adaptive responses to acute IRI in the heart may mobilize as yet unrecognized pathogenetic factors and help to identify novel therapeutic targets for cardioprotection. The hypoxia-inducible factors HIF-1α and HIF-2α orchestrate the body’s adaptive response to hypoxia (a major component of IRI) through the transcription and translation of several hundred proteins. While HIF-1α has been suggested as a therapeutic target for cardioprotection [35, 77], little is known about cardiac HIF-2α, which is involved in hypoxic signaling as well. Julian Aragonés (Spain) provided new information on the HIF-2α isoform, which acts as an mTORC1 activator via the amino acid carrier SLC7A5, especially promoting growth of renal cell carcinoma [19]. While several experimental studies have investigated the tissue protective role of HIF-2α in the brain [81], kidney [46], or skeletal muscle [5], respective studies in the heart have not been carried out yet. Since ischemia-provoked HIF-2α activation is essential for the transcription of new proteins that would take some time, therapeutic approaches appear to applicable more towards a delayed cardioprotection rather than to acute protection against IRI.

While hypoxia is known as a stimulus for the increase in sympathetic tone, observed in patients with arterial hypertension, Nephtali Marina (UK) presented evidence that the brainstem in hypertensive animals (spontaneously hypertensive rats, SHR) was relatively hypoxic as well, compared to normotensive Wistar rats [64]. As a consequence, increased levels of “ambient” ATP and lactate in the brainstem of SHR contributed to the development of arterial hypertension by increasing the activity of the pre-sympathetic circuits. Furthermore, it was shown that facilitated breakdown of extracellular ATP-modulated sympathetic activity [65] and attenuated the development of hypertension in SHR [64]. The importance of the Cushing reflex mechanism in the maintenance of adequate oxygen delivery to vital centers of the brain and its role in the pathogenesis of arterial hypertension was indicated as well, demonstrating the importance of glial cells in the regulation of sympathetic activity. Future studies are needed to clarify the metabolic and vascular mechanisms leading to the development of brainstem hypoxia in human subjects with arterial hypertension.

## Macrophage polarization as an anti-inflammatory therapeutic strategy

A variety of inflammatory processes has been shown to initiate and/or sustain molecular processes culminating in CVD [55, 80]. As such, the different steps of the inflammatory cascade as principal mechanism of innate immunity, of host defence and of tissue regeneration may persist or becomes dysregulated, resulting in chronic inflammatory disorders of the cardiovascular system, such a atherosclerosis [69]. In this regard, monocytes/macrophages respond to external stimuli with rapid changes in the expression of numerous inflammation-related genes to undergo polarization towards the M1 (pro-inflammatory), M2 (anti-inflammatory), or related sub-phenotypes [8, 13, 62]. Despite the absence of chitin, a polymer of β-N-acetyl-glucosamine in mammals [45], the chitin-cleaving glycosyl hydrolase chitotriosidase (Chitinase 1, CHIT1) is abundantly produced and secreted by activated macrophages in association with various human diseases. Importantly, elevated chitinase activity has been found in atherosclerotic lesions [50], and William Boisvert (USA) presented data that indicate a beneficial role for chitinase against vessel degeneration. In particular, the chitinase inhibitor allosamidin was shown to polarize macrophages towards the M1 phenotype to promote inflammation, indicative for the fact that chitinase may play a protective role in the pathogenesis of atherosclerosis by keeping macrophages in the M2 phenotype and promoting lipid uptake and efflux. Further investigations need to clarify the underlying protective mechanisms of chitinase and its possible use as a natural anti-atherogenic drug.

It has been recently shown that eRNA can exert pro-thrombotic and pro-inflammatory properties in the cardiovascular system and provoke cytokine mobilization as well as macrophage polarization, independent of Toll-like receptor-3 activation [91, 92]. Hector A. Cabrera-Fuentes (Germany and Singapore) demonstrated that mouse bone marrow-derived-macrophages (BMDM), which were differentiated with mouse macrophage-colony-stimulating factor into the M2-phenotype, were found to be skewed toward the inflammatory M1-phenotype when exposed to eRNA [8]. In accordance with the proposed actions of eRNA as a pro-inflammatory “alarm signal” and triggering factor for TACE, these data may shed light on the role of eRNA in the context of chronic inflammatory diseases such as atherosclerosis.

The bi-functional “Transient receptor potential melastatin-2” (TRPM2), which combines a nonselective calcium (Ca^2+^)-permeable channel with an adenosine diphosphate ribose pyrohydrolase activity [97], is activated by intracellular adenosine diphosphate ribose and allows both Ca^2+^ influx into the cell and/or Ca^2+^ release from lysosomes, contributing to cell migration, cell death, cytokine production, as well as degranulation in monocytes, dendritic cells, neutrophils, and mast cells [51, 73, 97, 98]. Adriana Sumoza-Toledo (Mexico) presented information on cardiac TRPM2, which is involved in inflammatory processes caused by oxidative stress, indicative for a role of this channel protein in protection against cardiac IRI [68] as well as for the maintenance of cardiac myocyte bioenergetics [39]. Thus, TRPM2 may also provide a potential target to regulate hyper-inflammatory processes in CVD.

## Targeting endothelial permeability as an anti-inflammatory therapeutic strategy

Endothelial junctional dynamics plays an important role not only in regulating endothelial permeability for a variety of solutes but also in controlling unwanted leukocyte emigration into tissues. A close interaction between the vascular endothelial (VE) -cadherin/catenin complex, the structural and functional backbone of endothelial cell junctions, and actin filament dynamics including actin polymerization and actin/myosin-mediated contractility, simultaneously maintains integrity and dynamics. Due to the lifelong shear stress-dependent biomechanical forces at specific sites of the vascular tree, the affected endothelial cells become continuously stimulated and acquire a dysfunctional phenotype that resembles the inflammatory endothelium. Such chronic conditions are associated with the disturbance of the vascular barrier properties and allow uncontrolled adhesion and transmigration of pro-inflammatory cells into the neointimal region of the inflammatory vessel wall to feed the atherogenic process. To follow the time-related changes and molecular arrangements of endothelial cell junction dynamics, life cell imaging using fluorescent tagged proteins has been very instrumental, as addressed by Hans Schnittler (Münster, Germany). Here, the formation of “Junction associated intermittent lamellipodia” (JAIL) has recently been reported to be one of the key mechanisms that control junction remodeling by largely maintaining cell-junction integrity [1, 2, 88]. JAIL are actin-driven and ARP2/3-N-WASP-controlled small plasma membrane protrusions appearing at gaps between VE-cadherin clusters, such that JAIL form new VE-cadherin adhesion plaques that cluster during JAIL retraction and subsequently become incorporated into the junctions. This process constitutively remodels VE-cadherin patterns and maintains endothelial integrity at the same time. This mechanisms also provides the driving force for cellular motility within a cell monolayer. In response to thrombin as a temporary pro-inflammatory factor, JAIL inhibition occurs with subsequent opening of endothelial cell junctions [90]. Although JAIL has been demonstrated to play a role in angiogenesis and involves the focal contact protein parvin [23], little is known about the relevance of such vascular cell connections in the cardiac macro- or microcirculation both under healthy conditions or in situations of IHD. It remains to be tested whether the identification of distorted cardiac JAIL may serve as marker of dysfunctional vascular endothelium and/or provide new targets for protective interventions.

## MicroRNAs, platelet dysfunction, and systems biology approach in cardiovascular research

Platelets from patients with diabetes are hyper-reactive and demonstrate increased adhesiveness, aggregation, degranulation, and thrombus formation as well as conjugate formation with leukocytes: processes that contribute to the accelerated development of vascular disease. Part of the problem seems to be dysregulated platelet Ca^2+^ signaling and the activation of intra-platelet calpains, which constitute Ca^2+^-activated proteases that are required for the limited proteolysis of several substrate proteins and subsequent alterations in platelet signaling. Ingrid Fleming (Germany) has found that the activation of µ- and m-calpain in patients with type 2 diabetes has profound effects on the platelet proteome and identified septin-5, the integrin-linked kinase (ILK) and Dicer as novel calpain substrates [18, 82]. The calpain-dependent cleavage of septin-5 disturbed its association with syntaxin-4 and promoted the secretion of α-granule contents, including TGF-β and the chemokine CCL5. The cleavage of Dicer in platelets from diabetic mice or patients thereby reduced the levels of platelet microRNAs miR-142, miR-143, miR-155, and miR-223. Deletion of miR-223 in mice resulted in modestly enhanced platelet aggregation, the formation of large thrombi, and delayed clot retraction compared to wild-type littermates. A similar dysregulation was detected in platelets from diabetic patients. Proteomic analysis of platelets from miR-223 knockout mice revealed increased levels of several proteins including kindlin-3 and coagulation factor XIII-A: While kindlin-3 was indirectly regulated by miR-223, factor XIII was a direct target and both proteins were also altered in diabetic platelets. Most importantly, treating diabetic mice with a calpain inhibitor for only 12 days prevented the loss of platelet Dicer as well as the diabetes-induced decrease in platelet microRNAs and normalized the platelet proteome as well as platelet function. Whether or not the activation of calpain and the subsequent cleavage of Dicer can account for changes in the microRNA profile associated with other diseases remains to be determined.

Finally, Manuel Mayr (UK) provided an overview from a systems biology perspective to help integrate the diverse topics in cardiovascular research for combined technological approaches in finding e.g., new biomarkers or causative relations. This can be achieved using proteomics in combination with other postgenomics technologies, such as lipidomics or microRNA profiling as well the integration of biological information in disease-specific networks for CVD. Unlike genomics, postgenomics approaches are not “off-the-shelf technologies” to identify novel biomarker candidates. Rather than evaluating individual biomarkers by routine clinical measurements, the linkage of cutting-edge postgenomics technologies with population studies aims to explore biomarker panels derived from a multi-omics approach (proteomics, lipidomics, and microRNA-omics) in a primary preventive setting [66, 95, 105, 106, 108, 110]. This integration of emerging technologies has the potential to improve our understanding of the etiology, prediction and stratification of CVD. As an primary example, this approach revealed the loss of platelet-related microRNAs in type 2 diabetes and could link a subset of platelet microRNAs (miR-126, miR-223, and miR-197) to cardiovascular risk in the prospective Bruneck study [108–110]. Consequently, such bioinformatic information may thus define new pathways where the unraveled molecular connections may be proven by in vitro experiments, by preclinical models as well as in clinical trials.

## Summary and conclusions

In summary, recent advances in cardiovascular research were discussed at this New Frontiers in Cardiovascular Research meeting covering a range of topics including cardioprotection, inflammation research, and vascular biology. A number of novel therapeutic targets for mediating cardiovascular protection in relation to a number of conditions including acute ischemia/reperfusion, heart failure, and atherosclerosis were introduced at the meeting. The important issue of translating new therapeutic strategies from the benchside to the bedside was highlighted given the challenges facing this process—these will have to be improved should we wish to impact on clinical outcomes in our patients with CVD.
